# Determinants of Sleepiness at Wheel and Missing Accidents in Patients With Obstructive Sleep Apnea

**DOI:** 10.3389/fnins.2021.656203

**Published:** 2021-04-13

**Authors:** Francesco Fanfulla, Gian Domenico Pinna, Oreste Marrone, Nadia D’Artavilla Lupo, Simona Arcovio, Maria R. Bonsignore, Elisa Morrone

**Affiliations:** ^1^Sleep and Respiratory Function Unit of the Pavia and Montescano Institutes, Istituti Clinici Scientifici Maugeri IRCCS, Pavia, Italy; ^2^Department of Biomedical Engineering of the Montescano Institute, Istituti Clinici Scientifici Maugeri IRCCS, Pavia, Italy; ^3^Istituto per la Ricerca e l’Innovazione Biomedica (IRIB), National Research Council (CNR), Palermo, Italy; ^4^Division of Respiratory Medicine, Dipartimento di Promozione Della Salute, Materno Infantile, Medicina Interna e Specialistica di Eccellenza “G. D’Alessandro” (PROMISE), University of Palermo, Palermo, Italy

**Keywords:** sleep apnea, OSA, excessive daytime sleepiness, sleepiness at wheel, COPD

## Abstract

**Study Objectives:**

Motor-vehicle crashes are frequent in untreated OSA patients but there is still uncertainty on prevalence as well as physiological or clinical determinants of sleepiness at the wheel (SW) in OSA patients. We assessed determinants of SW or sleepiness related near-miss car accident (NMA) in a group of non-professional drivers with OSA.

**Methods:**

A 237 consecutive, treatment-naïve PSG-diagnosed OSA patients (161 males, 53.1 ± 12.6 years) were enrolled. Self-reported SW was assessed by positive answer to the question, “Have you had episodes of falling asleep while driving or episodes of drowsiness at wheel that could interfere with your driving skill in the last year?” Occurrence of NMA in the last 3 years was also individually recorded. Habitual self-reported average sleep time was collected.

**Results:**

SW was found in 41.3% of patients but one-quarter of patients with SW did not report excessive daytime sleepiness. Predictors of SW were the following subjective factors: Epworth sleepiness scale score (ESS-OR 1.26; IC 1.1–1.4; *p* < 0.0001), depressive symptoms (BDI-OR 1.2; IC 1.06–1.18; *p* < 0.0001) and level of risk exposure (annual mileage-OR 1.9; IC 1.15–3.1; *p* = 0.007). NMAs were reported by 9.7% of patients, but more frequently by SW^+^ than SW^–^ (22.4% vs. 0.7%; χ^2^ 31, *p* < 0.0001). The occurrence of NMAs was significantly associated to ESS, BDI, habitual sleep duration and ODI (*R*^2^ = 0.41).

**Conclusion:**

SW is not predicted by severity of OSA. Evaluation of risk exposure, assessment of depressive symptoms, and reported NMA should be included in the clinical evaluation, particularly in patients with reduced habitual sleep time and severe nocturnal hypoxia.

## Introduction

Road traffic injuries are currently estimated to be the ninth leading cause of death across all age groups globally, and are predicted to become the seventh leading cause of death by 2,030 (World Health Organization, 2018).

Several studies suggested that sleepiness at the wheel (SW) is an important factor contributing to road traffic accidents: ten to 30 percent of fatal accidents have been attributed to SW (Horne and Reyner, 1999; Connor et al., 2002; Stutts et al., 2003; Sagaspe et al., 2010; Ohayon, 2012; Tefft, 2012; Philip et al., 2014; Bioulac et al., 2015). Moreover, this proportion of traffic accidents varies across countries from 3.9 to 33% in the United States, France, and New Zealand (Connor et al., 2002; Sagaspe et al., 2010; Tefft, 2012).

Sleepiness-related motor vehicle accidents have been acknowledged as resulting both from falling asleep while driving and from behavior impairment attributable to sleepiness (Dinges, 1995). Episodes of sleepiness at the wheel in the previous 2 years were reported by 17% of European drivers in a recent survey, underlining that it is a common problem (Goncalves et al., 2015). SW can be caused by various sleep disorders (i.e., sleep apnea) but also by behavioral factors such as sleep deprivation, shift work and non-restorative sleep (Goncalves et al., 2015; Bonsignore et al., 2016). However, the strength of the relationship between SW and motor vehicle accidents risk is very different among studies (Connor et al., 2002; Nabi et al., 2006; Philip et al., 2010, 2014; Sagaspe et al., 2010).

The correlation between OSA and motor vehicle accidents is confirmed by several studies reporting an increasing risk varying between two and seven-fold in comparison to the general population (McNicholas and Rodenstein, 2015). Motor vehicle crashes are more frequent in untreated patients and usually associated with more severe injuries (Mulgrew et al., 2008; Garbarino et al., 2016a). Regular CPAP treatment relieves excessive daytime sleepiness and reduces the crash risk (Marshall et al., 2006; Tregear et al., 2010; Karimi et al., 2015). However, it is a matter of discussion (McNicholas and Rodenstein, 2015) whether the driving risk in OSA is more closely related to the degree of daytime sleepiness or to the objective severity of sleep-disordered breathing. Excessive daytime sleepiness (EDS) in usually reported only by 30–50% of OSA patients (Ye et al., 2014; Saaresranta et al., 2016; Keenan et al., 2018) and there is no agreement about the main determinants of EDS (Punjabi et al., 1999; Mediano et al., 2007; Budhiraja et al., 2017; Prasad et al., 2018). At the same time, there is still uncertainty on prevalence as well as physiological or clinical determinants of sleepiness at the wheel in OSA patients. Masa et al. (2000) found that only one-half of habitually sleepy drivers reported overall EDS (defined as Epworth Sleepiness Scale score ≥9) and that breathing disorders during sleep were more frequent in habitually sleepy drivers (34%) than in controls (14%) considering an AHI cut-off of 5/h. However, in the habitually sleepy drivers the prevalence of sleep apnea was not different between subjects with or without automobile crashes. Recent systematic reviews and meta-analyses confirmed these findings (Garbarino et al., 2016b; Bioulac et al., 2017). In a large Swedish study, only severe EDS (ESS/ > 16), but not OSA severity, was associated with increasing risk of motor vehicle accidents (Karimi et al., 2015). Conversely, Arita and coll. reported an increasing frequency of sleepiness at wheel or car accidents in the previous 5 years in patients with severe or very severe OSA in comparison with mild to moderate OSA or simple snorer patients (Arita et al., 2015).

Near-miss sleepy car accidents (NMAs) are considered usual precursors of actual car accidents (Powell et al., 2007; Sagaspe et al., 2010), raising the need to know the determinants of NMAs in OSA patients. The present study was designed to assess the determinants of SW or NMAs in a group of non-professional drivers with OSA.

## Materials and Methods

### Patients

**FIGURE 1:**
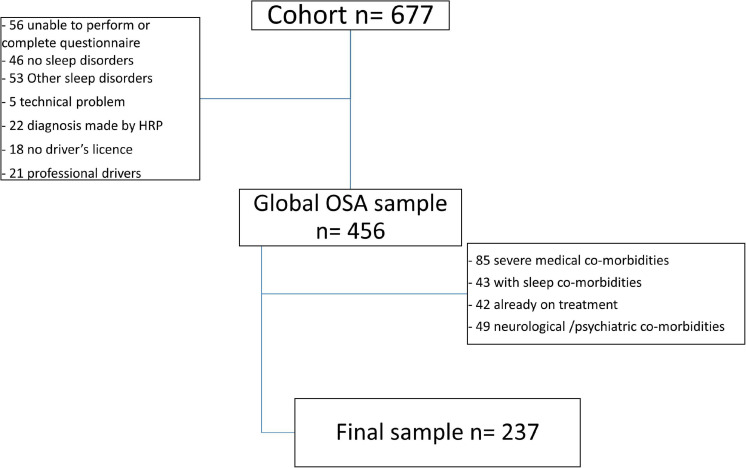
Flow diagram of patient selection. HRP, home respiratory polygraphy.

We enrolled 237 consecutive, treatment-naïve OSA patients (53.1 ± 12.6 years, 76 females) referred to our Sleep Unit for suspected OSA. Patients with another sleep disorder in addition to OSA, previous treatment for OSA, pregnancy, severe comorbidities (i.e., COPD, congestive heart failure, previous stroke) or regular assumption of drugs that could interfere with diurnal vigilance were excluded from the study as were professionals drivers (Figure 1).

### Sleep Evaluation

The diagnosis of OSA was made when international criteria were met (American Academy of Sleep Medicine, 2014). Structured clinical and sleep interview (McCarty, 2010) that includes a 47 items standard clinical questionnaire, Epworth sleepiness scale (ESS) (Johns, 1994), Pittsburgh Sleep quality index (PSQI) (Buysse et al., 1989), Insomnia Severity Index (ISI) (Bastien et al., 2001), Morningness-Eveningness Questionnaire (Adan and Almirall, 1991) and Beck depression inventory-II (BDI-II) (Beck et al., 1996) was administered to all patients. Similar to the study by Masa et al. (2000), excessive daytime sleepiness (EDS) was scored in the presence of an ESS ≥9.

Self-reported SW was assessed by positive answer to the question, “Have you had episodes of falling asleep while driving or episodes of drowsiness at wheel that could interfere with your driving skill in the last year?” Occurrence of NMAs in the last 3 years was also individually recorded. Regular alcohol consumption was defined as regular intake of at least 7 units/week.

Patients underwent home full-standard polysomnography (PSG) (Embla Titanium or MPR ST^+^ proxy device, Natus Inc., WI, United States) that was analyzed according to AASM rules (Adan and Almirall, 1991): the start and the end of the recording were planned on the basis of the patient’s sleeping habits. PSGs were manually analyzed by well trained technicians and subsequently reviewed by another investigator (FF). Hypopneas lasting at least 10 s were scored in the presence of a peak signal excursion drop by ≥30% of pre-event baseline using nasal pressure and were associated to a ≥3% oxygen desaturation and or an arousal. Classification of respiratory events (obstructive, central or mixed) was performed according to the above mentioned standard criteria (Berry et al., 2014).

Sleep habits, i.e., average sleep time, were self-reported. Estimated annual driving distance (km year^–1^) was determined for each patient. Annual driving distance was categorized in < or > 15,000 km^20^. Questions about SW, NMA sleep habits and estimated annual driving distance were part of the standard clinical questionnaire.

The study was approved by the ethical committee of the Clinical and Scientific Maugeri Institutes IRCCS (N. 2252 CE).

### Statistical Analysis

Data are presented as means ± SD or percentage. All the variables were checked for normality with the Shapiro-Wilk test. We used the Mann-Whitney test to assess differences between groups and the χ^2^ test to assess the association between SW or NMAs and gender, EDS, shift work. Simple and Firth’s multiple logistic regression analysis was performed to identify the risk factors associated with the development of SW or occurrence of NMAs. Multivariable models were developed using a backward elimination procedure after checking for the presence of collinearity between predictors. A *p* value < 0.05 was considered statistically significant.

Data were analyzed using STATISTICA version 10 (StatSoft Inc., 2011^1^) and SAS/STAT 9.4 (SAS Institute Inc., Cary, NC, United States).

## Results

Anthropometrics and sleep data are reported in Table 1. Only four patients referred episodes of car accidents in the 3 years before OSA diagnosis.

**TABLE 1:** Anthropometrics and sleep data in patients with or without sleepiness at wheel.

	**SW^+^*n* = 98**	**SW^–^*N* = 139**	**All groups *N* = 237**	***p***
**Age (yrs)**	52.1 ± 11.9	53.8 ± 13	53.1 ± 11.9	ns
**BMI (kg/m**^2^)	28.8 ± 8.1	29.3 ± 7.1	29.1 ± 7.5	ns
**Regular alcohol consumption (%)**	15.7	10.9	13.7	n.s.
**ESS**	9.7 ± 5	5.9 ± 3.5	7.5 ± 4.5	<0.0001
**Class annual mileage (%)**	17.6–50.5–31.9	27.8–54.8–17.4	23.5–53–23.5	0.02*
**ISI**	9.7 ± 5	5.9 ± 3.5	7.5 ± 4.6	<0.0001
**PSQI**	8.3 ± 5.5	6.7 ± 3.8	7.3 ± 4.6	<0.001
**MEQ-r**	16.2 ± 3.8	16.6 ± 3.8	16.4 ± 3.5	n.s.
**BDI-II**	10.7 ± 7.7	5.8 ± 6	7.8 ± 7.1	<0.0001
**Habitual Sleep Time (hr)**	6.3 ± 1.2	6.2 ± 1.5	6.3 ± 1.4	ns
**TST (min)**	358.1 ± 51.4	356.9 ± 45.5	357.3 ± 48	ns
**SE (%)**	83.8 ± 12	83.1 ± 10.7	83.4 ± 11.2	ns
**Sleep Onset (min)**	21.9 ± 27.4	17.2 ± 23.2	20 ± 25.8	ns
**N_1_ (%)**	11.7 ± 10.6	12.5 ± 12.6	12.1 ± 11.8	ns
**N_2_ (%)**	40.4 ± 12.7	39.7 ± 11.2	40 ± 11.8	ns
**N_3_ (%)**	25.8 ± 14.8	27.5 ± 11.8	26.8 ± 13.2	ns
**REM (%)**	22.8 ± 9.8	21.1 ± 7.7	21.7 ± 8.7	ns
Arousal Index (ev*hr^–1^)	38 ± 21.7	36.6 ± 22.1	37.2 ± 21.9	ns
AHI (ev*hr^–1^)	36.4 ± 27.3	32.3 ± 24.7	34 ± 25.8	ns
**T < 90%**	12.7 ± 20.9	12.8 ± 21.1	12.8 ± 20.9	ns
ODI (ev*hr^–1^)	26.7 ± 26.3	21.7 ± 22.2	20 ± 21.2	ns
**Mean desaturation amplitude (%)**	5.5 ± 3.3	4.5 ± 1.5	4.9 ± 2.4	0.001
LMI (ev*hr^–1^)	20.9 ± 25.7	16 ± 16.7	17.9 ± 21.1	ns
PLMI (ev*hr^–1^)	11.8 ± 21.6	7.1 ± 14	9 ± 17.7	ns

Class annual mileage: <10.000, 10.000–20.000 Km, >20.000 Km/year. *χ^2^ test.

### Sleepiness at the Wheel (SW)

**FIGURE 2:**
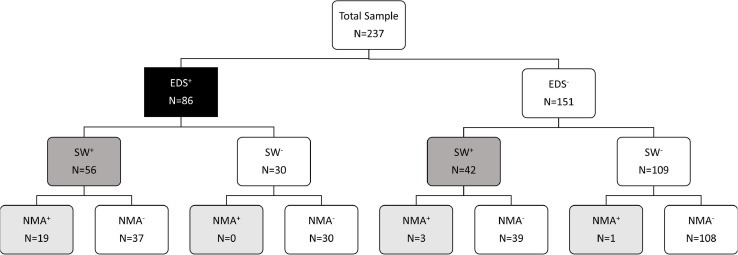
Flow diagram reporting distribution of patients according to presence of EDS, SW and NMA.

Sleepiness at the wheel was reported by 41.3% of patients, who also reported higher scores of ESS, PSQI, ISI, anxiety, and depressive symptoms, as shown in Table 1. No differences were found for age, sex, chronotype, drug use, OSA severity and objective sleep quality indices, while annual mileage was higher in SW^+^ than SW^–^ patients. A statistically significant association was found between SW and presence of EDS (χ^2^ 31.4, *p* < 0.0001): SW was present (SW^+^) in 65.1% of patients with EDS (EDS^+^) in comparison to 27.8% of patients without EDS (EDS^–^), as shown in Figure 2.

The association between SW and presence of EDS defined as ESS ≥10 was similar to that observed using a ESS cut-off 9 (χ^2^ 31.9, *p* < 0.0001): SW was present (SW^+^) in 70.1% of patients with EDS (EDS^+^) in comparison to 30% of patients without EDS (EDS^–^).

The occurrence of SW was significantly associated in univariate analysis to ESS score (OR 1.24; CI 1.15–1.34), BDI (OR 1.11; CI 1.05–1.17; *p* < 0.001), ISI (OR1.09; CI 1.04–1.14, *p* < 0.0001), PSQI (OR 1.08, CI 1.02–1.14, *p* = 0.01) and annual mileage (OR 1.0; CI 1.0–1.0, *p* = 0.004).

**FIGURE 3:**
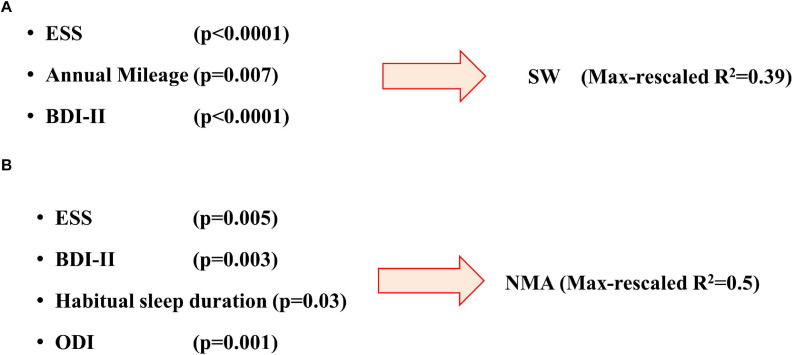
Predictive models for development of sleepiness at wheel **(A)** or occurrence of near-miss accidents **(B)**. Brackets report the *p* value of each predictive factor (see text for more details). ESS, Epworth Sleepiness Scale score; ODI, Oxygen desaturation index; BDI, Beck depression inventory-II score.

In multiple logistic regression analysis, depressive symptoms (BDI – OR 1.2; CI 1.06–1.18; *p* < 0.0001) ESS score (OR 1.26; CI 1.1–1.4; *p* < 0.0001) and annual mileage (OR 1; CI 1.0–1.0; *p* = 0.007) were independent predictors of SW (*R*^2^ = 0.39) (Figure 3A).

### Sleepiness Related Near-Miss Accidents (NMAs)

Overall NMAs were reported by 9.7% of patients, but their frequency was three-fold higher in SW^+^ than SW^–^ groups (22.4 vs. 0.7%; χ^2^ 31, *p* < 0.0001) as shown in Figure 2. Similarly, NMAs were reported more frequently by patients with (22.1%) than without EDS (2.6%, χ^2^ 23.6, *p* < 0.0001). Similar results were found when EDS was defined as ESS ≥10 (χ^2^ 31.4, *p* < 0.0001).

Near-miss sleepy car accidents^+^ patients showed higher score of ESS, ISI, PSQI, and BDI than NMAs^–^ patients (Table 2). Furthermore, comparison between NMAs^+^ and NMAs^–^ patients showed higher percentage of REM sleep, more severe sleep apnea and sleep fragmentation and a more severe sleep hypoxia in the NMAs^+^ group. No differences were found for age, sex, chronotype, shift work, alcohol or drug use.

**TABLE 2:** Anthropometric and sleep indices in patients with (NMA^+^) or without (NMA-) occurrence of near-miss accidents.

	**NMA^+^*N* = 23**	**NMA^–^*N* = 214**	**P**
**Age (yrs)**	51.4 ± 8	53.2 ± 13	ns
**BMI (kg/m**^2^)	30.1 ± 11	29 ± 7	ns
**Regular alcohol consumption (%)**	14.7	4.6	n.s.
**ESS**	11.5 ± 3.7	7.1 ± 4.5	<0.001
**Class annual mileage (%)**	21.1–42.1–36.8	23.7–54–22.2	n.s.
**ISI**	12.6 ± 6.5	8.4 ± 6.3	<0.01
**PSQI**	10 ± 6.4	7.1 ± 4.3	<0.01
**MEQ-r**	15.1 ± 3.3	16.5 ± 3.5	n.s.
**BDI-II**	13.9 ± 9.4	7.1 ± 6.6	<0.01
**Habitual sleep time (hr)**	5.8 ± 0.92	6.4 ± 1.4	ns
**TST (min)**	358 ± 28.8	357 ± 49.7	ns
**SE (%)**	85 ± 6.5	83.2 ± 11.6	ns
**Sleep onset (min)**	21 ± 23.8	19.9 ± 26.1	ns
**N_1_ (%)**	12.1 ± 10.7	12.2 ± 12	ns
**N_2_ (%)**	37.7 ± 12.6	40.2 ± 11.7	ns
**N_3_ (%)**	25 ± 16	27 ± 12.9	ns
**REM (%)**	25.1 ± 7.5	21.5 ± 8.7	0.05
Arousal index (ev*hr^–1^)	48.1 ± 23.1	35.9 ± 21.5	0.01
AHI (ev*hr^–1^)	48.5 ± 31.3	32.5 ± 24.8	<0.01
**T < 90%**	26.1 ± 30.2	11.3 ± 19.3	<0.01
ODI (ev*hr^–1^)	41 ± 30.1	21.8 ± 22.6	<0.001
**Average**	89.5 ± 2.8	89.7 ± 8.5	ns
LMI (ev*hr^–1^)	19.1 ± 21.2	17.7 ± 21.1	ns
PLMI (ev*hr^–1^)	8.6 ± 14.1	9.1 ± 18.2	ns

*Class annual mileage: <10.000, 10.000–20.000 Km, >20.000 Km/year.*

Within the group of patients with SW, those with NMAs reported lower habitual nocturnal sleep duration (5.5 ± 0.94 vs. 6.5 ± 1.2 h, *p* = 0.02), higher score of ESS (11.8 ± 3.4 vs. 9.1 ± 5.2; *p* = 0.02), ISI (13.1 ± 6.2 vs. 10.1 ± 6; *p* = 0.04), PSQI (10.3 ± 6.3 vs. 7.7 ± 5.1, *p* = 0.05) and BDI (14.6 ± 9.1 vs. 9.5 ± 7; *p* = 0.008), higher arousal index (47.2 ± 23.2 vs. 35.2 ± 20.7; *p* = 0.02) as well as higher AHI (47.9 ± 31.9 vs. 33.1 ± 25.1; *p* = 0.02), ODI (41.1 ± 30.8 vs. 22.3 ± 23.4; *p* = 0.02) and worse nocturnal hypoxia (T_90_ 26.9 ± 30.6 vs. 8.5 ± 14.9, *p* < 0.001).

All but one NMA^+^ patients reported SW. The occurrence of NMAs was significantly associated in univariate analysis to ESS score (OR 1.19; IC 1.095–1.3), BDI (OR 1.11; IC 1.05–1.17; *p* < 0.001), ISI (OR1.1; IC 1.03–1.17, *p* = 0.004), PSQI (OR1.11, IC 1.03–1.2, *P* = 0.008), Arousal index (OR 1.02, IC 1.005–1.04, *p* = 0.01), AHI (OR 1.02, IC 1.006–1.04, *p* = 0.006), and T_90_ (OR 1.02, IC 1.008–1.04, *p* = 0.003). However, in multiple logistic analysis only ESS, BDI, habitual sleep duration and ODI resulted as independent predictors of NMAs (Table 3 and Figure 3B).

**TABLE 3:** Risk factors for the occurrence of near-miss accidents in the entire sample.

	**Odds ratio**	**Lower CI**	**Upper CI**	***p***
ESS	1.16	1.04	1.28	0.005
Habitual Sleep Time	0.66	0.45	0.97	0.03
ODI	1.03	1.01	1.05	0.001
BDI	1.09	1.03	1.16	0.003

**R*^2^ = 0.41. c = 0.86. Multivariate logistic regression analysis. Dependent variable: Occurrence of NMA. CI: 95% confidence intervals. ESS, Epworth Sleepiness Scale score; ODI, Oxygen desaturation index; BDI, Beck depression inventory-II score.*

### Association With Validated Risk Factors

We assessed the relationship between SW or NMAs with previously reported risk factors in European Countries (Karimi et al., 2014): Epworth Sleepiness Scale ≥16; habitual sleep time ≤5 h; use of hypnotics; annual driving distance ≥15,000 km. At least one risk factor was identified in 47.2% of patients, while 18.1, 2.5, and 0.8% showed presence of 2, 3 or 4 risk factors, respectively. Differences in the distribution of risk factors between genders was close to statistical significance (χ^2^ 8.9; *p* = 0.061): 15.8% of females reported >2 risk factor in comparison to 24.2% of males. Habitual sleep time 5≤h was more frequent in females than males (30.3 vs. 18.1%, respectively, *p* = 0.03), while annual driving distance ≥15,000 km was more frequent in males than females (62 vs. 23.7%, respectively; *p* < 0.0001). Patients with habitual sleep time ≤5 h reported higher ISI, BDI, and PSQI score as well as reduced sleep efficiency and higher wake after sleep onset than those with habitual sleep time >5 h (Table 4); of interest, no differences were found in OSA severity, alcohol intake and in use of drugs.

**TABLE 4:** Differences between patients reported habitual sleep time duration ≤ or > 5 h/night.

**Habitual sleep time**	**≤5 h (*n* = 53)**	**>5 h (*n* = 184)**	***p***
**M:F**	30:23	131:53	0.04*
**Km/year**	21,308 ± 28.309	17,338 ± 16,753	n.s.
**AGE (yrs)**	51.4 ± 11.2	53.5 ± 12.9	n.s.
**ISI**	13.3 ± 5.7	7.5 ± 6	<0.0001
**PSQI**	11 ± 3.3	6.3 ± 4.4	<0.0001
**BDI**	10.2 ± 8.2	7.1 ± 6.7	0.005
**Drugs (%)**	26.5	16.9	n.s.*
**Alcohol (%)**	6.3	15.7	n.s.*
**ESS**	7.2 ± 4.5	7.5 ± 4.5	n.s.
**WASO (min)**	65.6 ± 50.6	47.4 ± 36.7	0.004
**SO (min)**	18.6 ± 20.3	20.5 ± 27.3	n.s.
**SE (%)**	80.5 ± 12.5	84.3 ± 10.8	0.03

*χ2 test.

A statistically significant association was found between prevalence of risk factors and development of sleepiness at wheel (χ^2^15.8; *p* = 0.003) but not with occurrence of NMAs (p n.s.): NMAs were reported by 9.5% of patients without any risk factors.

In a multiple logistic regression analysis model, severity of depressive symptoms (OR 1.1; IC 1.06–1.16; *p* ≤ 0.0001) and number of risk factors (OR 1.66; IC 1.15–2.38, *p* = 0.006) were predictors of SW. On the contrary, number of risk factors were not predictive of NMAs.

## Discussion

The main findings of the present study are:

(1) SW^+^ was observed in 98 patients (41.3% of total sample), but one quarter of them did not report excessive daytime sleepiness, while NMAs were reported by 23 patients (9.7% of total sample).
(2) Predictors of SW in OSA patients were subjective factors, EDS, presence of depressive symptoms and level of risk exposition (annual mileage).
(3) Severity of nocturnal hypoxia contribute with reduced habitual sleep time and excessive daytime sleepiness in determining NMAs.

Sleepiness at wheel can be caused by various sleep disorders including OSA, but also by behavioral factors such as sleep deprivation, shift work, and non-restorative sleep. SW is frequent in the general population: in a recent survey 17% of European drivers reported episodes of sleepiness at wheel in the previous 2 years (Goncalves et al., 2015). However, prevalence was highly variable across the studies, according to different definitions of SW, different age distribution, percentage of females or professional drivers (Bioulac et al., 2017). A 3.6% of habitual sleepy drivers or drowsy drivers were reported by Masa in the general population (Masa et al., 2000); Maia et al. (2013) reported similar findings in a large population of drivers (Beck et al., 1996). Furthermore, considering the studies that used a SW definition similar to the one used in our study, prevalence of SW ranged from 25.1 to 57.8% (Bioulac et al., 2017). Surprisingly, data on SW prevalence in OSA patients are scarce. The percentage of patients with SW in the present study was quite similar to that reported by Matsui et al. (2017) in a retrospective analysis involving 161 OSA patients, predominantly males.

Sleepiness at the wheel is more frequent in OSA patients with concomitant EDS, but more than one quarter of patients in the present study reported SW without concomitant EDS. These results are in line with the study of Masa where one-half of habitually sleepy drivers reported sleepiness occurring predominantly during driving, and with data reported in the meta-analysis of Bioulac (Bioulac et al., 2017). All these data reinforce the idea that sleepiness at wheel is a specific task that should be assessed independently of excessive daytime sleepiness. Teran-Santos et al. (1999) found a strong association between sleep apnea and risk of traffic accidents, but the Epworth Sleepiness Scale failed to identify subjects with a higher risk of accidents. Ellen et al. (2006) analyzed and reviewed 40 relevant studies and concluded that drivers with OSA are at risk of involvement in motor vehicle crashes. However, an association between crash risk and daytime sleepiness or severity of sleep apnea was found only in a minority of studies, so that the authors stated “clinicians should be cautious in using the presence or absence of this symptoms as the sole factor in determining drive performance in patients with sleep apnea.”

Development of SW in OSA cannot be predicted by the polysomnographic variables describing OSA severity, i.e., AHI, nocturnal hypoxia and sleep fragmentation, alone or in combination. Instead, presence of EDS, amount of depressive symptoms and exposition to risk (annual mileage) predicted SW. Prevalence of SW found in the present study are similar to that recently reported by Philip et al. (2020) in a large cross-sectional analysis of French national OSA registry. They found that patients reporting sleepiness at the wheel, whatever their obstructive sleep apnoea status and severity, exhibited a ten-fold higher risk of sleepiness-related accidents.

Fatigue or sleep-related accidents represent a common cause of traffic accidents in industrial societies (Horne and Reyner, 1999; Philip et al., 2001; Connor et al., 2002; Sagaspe et al., 2010; Fanfulla et al., 2013), but the proportion of accidents related to sleepiness varies across countries. Car crashes related to falling asleep are likely to cause death or severe injury: it has been estimated that fatality occurs in 11.4% of sleepiness-related accidents in comparison to 5.6% of general accident (Garbarino et al., 2001).

In the present study sleepiness-related near-miss accidents were reported by ten percent of patients, almost all of them with sleepiness at wheel. Similar results were reported by Philip et al. (2014) in a population-based case-control study. Overall, patients with NMAs showed higher severity of OSA, both in terms of AHI and nocturnal hypoxia, higher sleep fragmentation, clinical symptoms including higher score of Epworth Sleepiness scale. The association between OSA and increased risk of motor vehicle crash was the aim of a systematic review and meta-analysis by Tregear et al. (2010). The authors analyzed eleven studies reporting conflicting results: in three of them a trend toward greater severity of OSA among individuals who crashed was found. In three other studies severity of OSA was associated with an increased risk of motor vehicle crash, but in the remaining five studies this association was not confirmed. Our data are coherent with the finding of Ward et al. (2013). They reported in a large population of sleep clinic patients an association between OSA severity or EDS with the rate of NMAs and an important gender effect: severe OSA was protective for near-misses or crash occurrence in females, and the association between EDS and near-misses or crash was mild or absent. In the present study we did not find any gender effect both for development of sleepiness at wheel or for near miss accidents. A possible explanation of these discrepancies may be related to different method used for data collection: in their study, Ward et al. (2013) included self-reported near misses or crash data over the entire driving history of the single patients and compared data with the level of EDS or OSA severity measured at the moment of the sleep evaluation. We used data temporally limited to the year preceding OSA diagnosis for sleepiness at wheel, and to the last 3 years for NMAs: in this way the association with OSA or EDS severity as well as other known potential risk factors (sleep log; work schedules etc.) appeared more consistent, limiting also recall or report bias.

While the relationship between OSA, EDS, and depression is known (Mokhlesi et al., 2016; Bonsignore et al., 2019) only few studies were focused on the presence of sleepiness at wheel, motor vehicle crashes and depressive symptoms with conflicting results. Nabi et al. (2006) in a large sample of employees of the French national electricity and gas company found an increasing risk of sleepiness at the wheel in subjects with depressive symptoms. Philip et al. (2014) found that subjects on treatment with anti-depressant or subjects with anxiety symptoms had higher risk of being involved in a road traffic accident. We obtained similar results in a population of OSA, patient since depressive symptoms were independent risk factors for development of SW or near accidents. However, Barbé et al. (1998) in a case control study did not find any correlation between risk of automobile accident and degree of anxiety or depression in OSA patients.

We confirmed the role of short habitual sleep time as a risk factor for NMA, as previously reported in general population or in patients with OSA (Powell et al., 2007; Berry et al., 2014; Philip et al., 2014; Sunwoo et al., 2017). Overall sleep restriction may be due to works schedules, shift work, social jet lag but may also be expression of insomnia, as a sleep-comorbidity or of a “insomnia-like OSA phenotype.” Indeed, it is now well recognized that about one third of OSA patients reported insomnia like-symptoms (Saaresranta et al., 2016). In the present study, patients with short sleep time reported higher Insomnia Severity Index or PSQI, prolonged WASO and reduced sleep efficiency, supporting the hypothesis of insomnia phenotype. We did not find differences in ISI, PSQI or Epworth score, as well as sleep efficiency between patients according to chronic use of drugs.

### Limitations of the Study

We included self-reported data both for SW and NMA that may potentially underestimate their prevalence and limits the strength of association with OSA severity indices. These biases have been previously discussed (Bioulac et al., 2017): an objective assessment of sleepiness at wheel would require specific and time-consuming tests that cannot be performed in a clinical setting and in a large number of patients. Another limitation is the lack of information about the time of day when the near miss accidents occurred. NMAs are self-reported in nature. However, data were collected at the time of the first clinic evaluation together with those of standard sleep questionnaire reducing the possibility for the patients to bias the answer. Furthermore, we limited the data of SW and NMA, respectively, to the last year or the last 3 years to avoid any recall bias.

We took care in excluding patients with another sleep disorder in addition to OSA or with severe comorbidities to avoid possible confounding effects with regard to the role of OSA *per se* in determining SW.

In conclusion, we suggest that a specific path for identification and assessment of OSA patient at risk for motor vehicle accident can be proposed: a specific question for SW should be added in the standard clinical evaluation as already suggested by Bioulac et al. (2017); together with evaluation of risk exposition (annual mileage); assessment of depressive symptoms; reported NMA, particularly in patients with reduced habitual sleep time and severe nocturnal hypoxia. Prioritization of these patients to diagnostic and therapeutic assessment may reduce the risk of motor or work accidents (Bonsignore et al., 2019).

## Author’s Note

Sleepiness at wheel is not predicted by severity of OSA. Other factors should be considered: presence of excessive daytime sleepiness, presence of depressive symptoms and level of risk exposure (annual mileage). The habitual sleep duration and the severity of nocturnal hypoxia resulted as the only significant predictors of near miss sleepy car accidents.

## Data Availability Statement

The data underlying this article will be shared on reasonable request to the corresponding author.

## Ethics Statement

The studies involving human participants were reviewed and approved by the Ethical Committee of the Clinical and Scientific Maugeri Institutes IRCCS (N. 2252 CE). Written informed consent for participation was not required for this study in accordance with the national legislation and the institutional requirements.

## Author Contributions

FF: study design. ND’A, EM, SA, and FF: data collection. FF and GP: data analysis and interpretation. FF and GP: literature search and generation of figures. FF, GP, OM, and MB: manuscript preparation. FF, GP, OM, and MB: manuscript drafting. All authors approved the final version of the manuscript.

## Conflict of Interest

The authors declare that the research was conducted in the absence of any commercial or financial relationships that could be construed as a potential conflict of interest.

## Footnotes

1 www.tibco.com
